# From laboratory to field: laboratory-measured pesticide resistance reflects outcomes of field-based control in the redlegged earth mite, *Halotydeus destructor*

**DOI:** 10.1007/s10493-023-00787-2

**Published:** 2023-03-31

**Authors:** Paul A. Umina, Leo McGrane, Joshua A. Thia, Evatt Chirgwin, Ary A. Hoffmann

**Affiliations:** 1 Cesar Australia, 95 Albert St, Brunswick, Victoria 3056 Australia; 2 School of Biosciences, Bio21 Institute, University of Melbourne, Victoria, 3010 Australia

**Keywords:** Organophosphate, Field control, Insecticide resistance, Bioassay, Omethoate, Chlorpyrifos

## Abstract

Resistance to pesticides is typically identified via laboratory bioassays after field control failures are observed, but the results of such assays are rarely validated through experiments under field conditions. Such validation is particularly important when only a low-to-moderate level of resistance is detected in the laboratory. Here we undertake such a validation for organophosphate resistance in the agricultural pest mite *Halotydeus destructor*, in which low-to-moderate levels of resistance to organophosphorus pesticides have evolved in Australia. Using data from laboratory bioassays, we show that resistance to the organophosphate chlorpyrifos is higher (around 100-fold) than resistance to another organophosphate, omethoate (around 7-fold). In field trials, both these chemicals were found to effectively control pesticide-susceptible populations of *H. destructor*. However, when applied to a resistant mite population in the field, the effectiveness of chlorpyrifos was substantially decreased. In contrast, omethoate remained effective when tested alone or as a mixture with chlorpyrifos. We also show that two *novel* (non-pesticide) treatments, molasses and wood vinegar, are ineffective in controlling *H. destructor* when sprayed to pasture fields at rates of 4 L/ha. These findings suggest a close link between levels of resistance quantified through laboratory bioassays and the field effectiveness of pesticides; however, in the case of *H. destructor*, this does not necessarily mean all field populations possessing organophosphate resistance will respond similarly given the potentially complex nature of the underlying resistance mechanism(s).

**Supplementary Information:**

The online version contains supplementary material available at 10.1007/s10493-023-00787-2.

## Introduction

Resistance to pesticides in insect and mite pests is typically detected following sub-optimal control being observed under field conditions. Although many chemical failures are the result of application issues, repeated control failures – followed by bioassays under controlled laboratory conditions – will often lead to resistance being identified as the underlying cause (Subramanyam and Hagstrum 1996; Picollo et al. 2005). Where laboratory bioassays indicate a high level of resistance, such that individuals with resistance can survive pesticide doses that are several thousand times higher than their susceptible counterparts, a high level of confidence can be attributed to the causal connection between field control failures and bioassay results. However, in other cases, the level of apparent resistance in individuals can be much lower, in the range of < 10 to a few hundred times higher than susceptible individuals (Siqueira et al. 2000; Kristensen and Jespersen 2003; Picollo et al. 2005; Tiwari et al. 2011). Where resistance levels are low or unclear, interpretations of impact may be difficult (Dennehy et al. 1987; Bagi et al. 2015) and it becomes important to link the presence of resistance to putative control failures. A low level of resistance may indicate an underlying mechanism that provides a relatively weak level of resistance (Picollo et al. 2005) but also a low frequency of resistant genotypes in a given population, which can be further tested through selection experiments (Snodgrass 1996; Kristensen and Jespersen 2003; Alvi et al. 2012).

In this paper, we evaluate the connection between laboratory-based resistance assays and field resistance in the redlegged earth mite, *Halotydeus destructor* (Tucker), a damaging mite pest of winter grains and pastures in several countries. In Australia, where we conducted our research, this species is widely distributed across the southern grain-growing regions, which have a Mediterranean-type climate (Wallace and Mahon 1971; Ridsdill-Smith 1997). *Halotydeus destructor* is a winter-active pest, with a winter egg phase from approximately April to November, during which time mites reproduce sexually, having up to four generations per year in Australia (Ridsdill-Smith 1997; Umina and Hoffmann 2004). Mites survive the dry, hot conditions of summer as diapause eggs in the cadaver of female mites, which hatch the following autumn when temperatures and rainfall are adequate (Ridsdill-Smith 1997; McDonald et al. 2015). *Halotydeus destructor* is particularly damaging at the establishment phase of crops and pastures when plant seedlings are most vulnerable to mite attack (Ridsdill-Smith 1997).

Chemicals remain the major way in which *H. destructor* is managed, although there are only five chemical Mode of Action (MoA) groups currently registered for control of *H. destructor* in Australia (APVMA, 2022). Synthetic pyrethroids (SPs) and organophosphates (OPs) are the two most frequently used chemicals and are primarily applied to crops and pastures as foliar sprays (Umina et al. 2019; Arthur et al. 2021). Neonicotinoid-based seed treatments are also used to protect emerging seedlings from *H. destructor* feeding in several winter crops, particularly canola (*Brassica napus*) (Ridsdill-Smith et al. 2008). The long-term and heavy reliance on chemicals, combined with the high densities that *H. destructor* populations can reach (Ridsdill-Smith et al. 2008), has led to the evolution of pesticide resistance. First discovered more than 15 years ago (Umina 2007), resistance has become an increasing threat to farmers in the southern growing regions of Australia (Maino et al. 2018). Resistance to both SPs and OPs now spans large parts of Western Australia and South Australia, with OP resistance also recently discovered in Victoria (Arthur et al. 2021). Additionally, there are populations of *H. destructor* that display dual resistance to both SPs and OPs, further reducing the number of options available to manage *H. destructor* effectively.

The expression of resistance measured through laboratory bioassays can be quite different to that in the field, which makes it difficult to predict how resistance in *H. destructor* is likely to influence the dynamics of field populations. Laboratory environments are artificial and often present the ‘ideal’ conditions when exposing potentially resistant mites to chemicals in order to assess pesticide responses. In the field, mites will experience differences in exposure to pesticides due to a multitude of factors, such as varying climatic conditions, interactions between plant chemistry and pesticides, longer exposure periods and fitness costs associated with resistance (Guillebeau et al. 1989; Tabashnik et al. 2003; Desneux et al. 2007; Bock et al. 2015). Furthermore, mite populations are typically under more stress than during standardised laboratory conditions, which can impact both survival and chemical response (e.g., Cahill et al. 1996, Heye et al. 2019). It is critical that assessments of the presence and levels of resistance under laboratory conditions can be meaningfully extrapolated into relevant recommendations for management in the field (Umina et al. 2019).

In *H. destructor*, the magnitude of SP resistance is extremely high, with laboratory bioassays indicating resistance ratios of up to 240,000-fold for bifenthrin and almost 60,000-fold for alpha-cypermethrin (Umina 2007). This resistance is conferred by knockdown resistance (*kdr*) mutations in the voltage gated para-sodium channel (Edwards et al. 2018), a mechanism that has evolved repeatedly in many arthropod pests in response to chemical exposure (Soderlund 2008; Van Leeuwen et al. 2010). Unsurprisingly, farmers have reported poor levels of control when using pyrethroid chemicals on mites with *kdr* resistance, even when applying multiple SP sprays (Umina 2007). Field experiments have also shown applications of pyrethroid chemicals to SP-resistant mites to be completely ineffective and, in fact, these can rapidly increase the frequency of SP resistance in mite populations (Cheng et al. 2021). Conversely, mites exhibiting OP resistance exhibit relatively low-to-moderate levels of resistance, with laboratory bioassays indicating resistance ratios ranging from ~ 5-400-fold. The variability in OP resistance that has been observed in previous studies is dependent on the active ingredient and/or the mite population tested (Umina et al. 2017; Maino et al. 2018; Arthur et al. 2021). The mechanism conferring OP resistance in *H. destructor* has not yet been elucidated, although it is likely to be a polygenic trait linked to acetylcholinesterase, the target site of organophosphates and carbamates (Thia et al. 2022a). Moreover, there have been no experiments that have directly investigated how OP resistance characterised in the laboratory corresponds to chemical efficacy against mites in the field.

The aim of this study was to examine the relationship between laboratory-based measures of OP resistance for *H. destructor* and the efficacy of chemical control in the field. Using previously collected bioassay data, we characterised the magnitude of resistance to two OP active ingredients that are widely used to control *H. destructor* in Australia. We also confirmed the absence of resistance to SPs in this population by screening for *kdr* mutations known to confer resistance. We then performed a large-scale replicated spray trial to examine how mite abundances are affected following OP exposure in the field. We also explored the impact of mixing two OP active ingredients. Because there are so few pesticide options available to manage *H. destructor*, we additionally tested the efficacy of two *novel* chemical treatments as part of the field trial. Our results have important management implications and shed further light on the value of laboratory-based methods when conducting resistance surveillance for mite pests.

## Materials and methods

### Pesticide laboratory bioassays

In a previous experiment, we undertook a series of laboratory bioassays to understand how temperature affects the chemical response of *H. destructor* (see Thia et al. 2022b). As part of this study, mites were collected from a field known to contain OP resistance (herein referred to as ‘Res-pop’) and from a nearby field known to contain mites that are susceptible to OPs (herein referred to as ‘Sus-pop’). In the current study, we re-analysed a sub-set of the laboratory bioassay data from Thia et al. (2022b) and so briefly summarise the relevant methods below and detail the statistical analysis we undertook.

Following collections between June and August 2021, mites were transported back to the laboratory, and subsequently tested over a series of blocked experiments against two OPs, omethoate (Le-mat 290 SL; Arysta LifeScience, Adelaide, Australia) and chlorpyrifos (Lorsban 500 EC; Corteva Agriscience, Chatswood, Australia). The recommended field rates for these chemicals, assuming an application in 100 L water/ha, were 290 mg a.i./L for omethoate and 700 mg a.i./L for chlorpyrifos. A coated vial bioassay was used to expose mites from both populations to eight doses of each pesticide (10×, 1×, 0.01×, 0.005×, 0.001×, 1e-4×, 1e-5×, and 1e-6× the field rate), along with a control of water.

For the purposes of the current study, we were only interested in those treatments where mites were held at 4 °C prior to testing and incubated at 18 °C during pesticide exposure (i.e., acclimation treatment = ‘Acclim cool’ and test treatment = ‘Test warm’; see Thia et al. 2022b). This is the standard methodology for pesticide laboratory bioassays involving *H. destructor* (Hoffmann et al. 1997; Umina 2007; Maino et al. 2018). We re-analysed these chemical response data via binomial logistic regression models using the lme4 package in R (Bates et al. 2015). The response variable ‘mite mortality per vial’ was adjusted for control mortality with Abbotts’ correction (Abbott 1925). We included ‘pesticide dose’ and ‘population’ as fixed effects and ‘experimental block’ as a random effect. We included an observation-level random effect to account for overdispersion (Elston et al. 2001). For each model, we first tested whether populations had overall differences in mortality (i.e., model intercept) by comparing the change in model deviance (χ^2^ tests). Next, we tested whether populations had differences in mortality that were dependent on pesticide dose (i.e., differences in regression slopes) by comparing the change in model deviance when all populations were constrained to have the same intercept (i.e., additive predictors only) and when each population had its own slope (i.e., inclusion of population × dose predictor term). We subsequently used the full model (additive + interactive predictors) to estimate the doses that resulted in 50% mortality (lethal concentration, LC) (along with 95% confidence intervals, CIs) using the binomial error distribution. We used these values to estimate the magnitude of resistance (resistance ratios) by dividing the LC_50_ value of the Res-pop by the LC_50_ value of the Sus-pop for each active ingredient.

### Genetic screening of *kdr* resistance

The status of SP resistance in both the Res-pop and Sus-pop was assessed by screening for the two *kdr* mutations (L1024F polymorphisms: TTG/TTC and TTG/TTT) known to confer SP resistance in *H. destructor* (Edwards et al. 2018). Our DNA extraction and TaqMAN SNP genotyping assays followed the methods described by Arthur et al. (2021). In total, we screened 50 mites from each population, and in both instances, only detected homozygous susceptible (i.e., TTG/TTG) individuals.

### Field experiment

#### Trial design and chemical treatments

To understand the relationship between laboratory-measured resistance in *H. destructor* and mite dynamics in the field, we undertook a field trial involving the same OP-resistant mite population (Res-pop) tested in the laboratory bioassays described earlier. This field site was an irrigated, long-term pasture field, consisting mostly of annual ryegrass (*Lolium multiflorum*) and subclover (*Trifolium subterraneum).* Historically, this field has repeatedly been treated with OPs, and more recently, with SPs. A large-scale field trial was established in the spring of 2021, which is the time of the year when *H. destructor* reach high population sizes and can be very damaging to pasture plants (Ridsdill-Smith et al. 2008). The trial consisted of seven treatments (Table 1), spread across 40 plots in a randomised design. The untreated control and four pesticide treatments were replicated 6× (30 plots) and the two *novel* treatments were replicated 5× (10 plots). Plots measured 20 × 20 m, which is equal to or larger than plot sizes used in previous field trials involving *H. destructor* and other earth mite species (e.g., Arthur et al. 2013, Jenkins et al. 2013, Umina et al. 2015).

**Table 1:** List of treatments applied at the field trial in 2021

Treatment	Chemical product	Active ingredient (a.i.)	Field application rate
Untreated control	--	--	--
Chlorpyrifos	Lorsban® 500 EC	Chlorpyrifos 500 g/L	700 mg a.i./ha
Omethoate	Le-mat® 290 SL	Omethoate 290 g/L	290 mg a.i./ha
OP Mix	Lorsban® 500 EC + Le-mat® 290 SL	Chlorpyrifos 500 g/L + omethoate 290 g/L	700 mg a.i./ha + 290 mg a.i./ha
Bifenthrin	Talstar® 250 EC	Bifenthrin 250 g/L	100 mg a.i./ha
Wood vinegar	Elmore Compost and Organics Wood Vinegar	Chicken litter and rice hull biochar by-product	4 L/ha
Molasses	Brandon Molasses	Blackstrap sugar cane molasses	4 L/ha

Of the pesticide treatments, we examined omethoate and chlorpyrifos, which matched the active ingredients tested using the pesticide laboratory bioassays. A mix of omethoate and chlorpyrifos (herein referred to as ‘OP Mix’) was also tested, given chemical mixtures can be an effective resistance management strategy. Additionally, we included the SP bifenthrin (Talstar 250 EC; FMC Australia, North Ryde, Australia) as a positive control. This chemical is regularly used to protect pastures and grain crops from *H. destructor* across southern Australia. All treatments were directly applied at the registered field rates for *H. destructor*, as per the label instructions (APVMA 2022). For the OP Mix treatment, the field rate of each active ingredient (chlorpyrifos and omethoate) was used. We included an untreated control (field plots that received no chemical application) as a negative control.

We also investigated the efficacy of two *novel* treatments: molasses (Brandon Molasses, West Melbourne, Australia) and wood vinegar (Elmore Compost and Organics, Elmore, Australia). These were chosen based on previous studies and industry reports, as well as through discussions with local farmers who had reported some success in controlling *H. destructor* with these products. Previous laboratory experiments and field trials in raspberries by Shanks et al. (1995) documented some degree of control when using molasses on the two-spotted spider mite (*Tetranychus urticae)*. Other studies have indicated molasses can reduce springtail (*Onychiurus* spp.) numbers in sugar beet (Heijbroek et al. 1980). Wood vinegar is a by-product of the biochar process, with some wood vinegar formulations showing potential to control arthropod pests, including termites (*Reticulitermes speratus*) (Yatagai et al. 2002) and the cowpea weevil, *Collosobruchus maculates* (Chalermsan and Peerapan 2009).

All treatments were applied as foliar sprays in a total volume of 100 L/ha at 300 kPa pressure using a trailing boom sprayer (UniBoom model 600 L TR, with TeeJet Flatfan nozzles [02-Fine]) to mimic field spraying conditions. To maximize spray efficacy, all treatments were applied in dry conditions when average wind speed was below 15 km/h.

#### Mite abundance assessments

Mite sampling closely followed the methodologies used in previous field trials involving *H. destructor* (e.g., Jenkins et al. 2013, Arthur et al. 2014, Cheng et al. 2021). The abundance of mites within each field plot was assessed prior to chemical treatment and again at 3, 7, 14 and 21 days after treatment (DAT). A petrol-driven blower vacuum (SH86; Stihl, Germany) with a 100-micron steel sieve fitted to the end of the vacuum was used to sample mites. A 30 × 30 cm wooden quadrat was placed randomly within the sample area of each plot and the nozzle of the suction sampler moved over the soil surface and plant material in this area for 10 s. This was repeated 4× in each plot. By using a defined quadrat, we ensured the sampling area was constant and mite counts could be extrapolated to individuals per m^2^. All vacuum sampling occurred within the inner 10 × 10 m of each plot, ensuring there was a 10 m buffer around each sample area to reduce any potential impacts of spray drift and/or mite movement from surrounding plots. The contents of each vacuum sample were dispensed into a plastic tray and all mites were directly counted.

### Statistical analysis

We used a mixed effects model with repeated measures to determine how treatments applied in the field trial affected mite abundance. Our model took the form: abundance = treatment + sampling date + treatment: sampling date + plot.

The response ‘abundance’ is the log(*n* + 1)-transformed number of mites per m^2^ based on observed numbers in quadrats. Abundance was modelled as a function of ‘treatment’, a categorical variable for treatments applied to plots, ‘sampling date’, a continuous variable for the number of days after treatment, and ‘treatment: sampling date’, their interaction. The random effect ‘plot’ allowed for unique intercepts for each treated plot. The model was fitted using the lmer function from the lme4 package in R (Bates et al. 2015). Significance of model terms was assessed using the Anova function from the car package in R (Fox and Weisberg 2018).

Post hoc comparisons were performed to assess differences among treatments at each sampling date. To do this, we fitted individual mixed effect models for each sampling date separately, such that: abundance = treatment + plot.

We then used Tukey’s tests to compare the mean differences among treatments within each sampling date using the multcomp (Hothorn et al. 2008) and multcompView (Graves et al. 2019) packages in R. Although mite abundances were transformed prior to analyses, we plotted untransformed values to maintain biological meaning.

## Results

### Dose-response relationships from laboratory bioassays

Full dose-response curves for *H. destructor* populations to chlorpyrifos and omethoate are shown in Figure S1. Control mortality was < 10% in both populations to each active ingredient and the curves closely match those found in other studies (Umina 2007; Maino et al. 2018; Arthur et al. 2021). Differences in responses were detected between the Res-pop and Sus-pop to both omethoate and chlorpyrifos. For both active ingredients, dose-response curves for the Res-pop were shifted to the right of the Sus-pop (Figure S1). There were significant population differences in responses to chlorpyrifos (χ^2^ = 24.46, df = 1, *P* < 0.001) and omethoate (χ^2^ = 5.81, df = 1, *P* = 0.02). LC_50_ values further confirm differences in responses between the Res-pop and Sus-pop (Table 2). For chlorpyrifos, there were non-overlapping 95% CIs between populations, and LC_50_ values for the Res-pop and Sus-pop were 14.90 and 0.15 mg a.i./L, respectively. This equates to a resistance ratio of ∼99 for chlorpyrifos. For omethoate, LC_50_ values for the Res-pop and Sus-pop were 1.99 and 0.30 mg a.i./L, respectively, equating to a resistance ratio of ∼7 (Table 2).

**Table 2:** LC_50_ values (95% CIs in parentheses) and mean regression coefficients (± SE) for *Halotydeus destructor* from the Res-pop and Sus-pop after 24 h exposure to chlorpyrifos and omethoate

Active ingredient	Population	No. mites tested	LC_50_ value (95% CIs) mg a.i./L^1^	Regression coefficient (± SE)	χ^2^ (df = 1)	*P*	Resistance ratio
Chlorpyrifos	Res-pop	525	14.90 (1.66-133.74) a	0.61 ± 0.09	24.46	< 0.01	99.3
	Sus-pop	456	0.15 (0.04–0.50) b	1.30 ± 0.24			
Omethoate	Res-pop	569	1.99 (0.65–6.12) a	0.77 ± 0.10	5.81	0.02	6.6
	Sus-pop	449	0.30 (0.11–0.82) b	1.26 ± 0.61			

^1^ Different letters represent significant differences between populations within each active ingredient (Tukey’s test: *P* < 0.05)

### Chemical control in the field

**Fig. 1:**
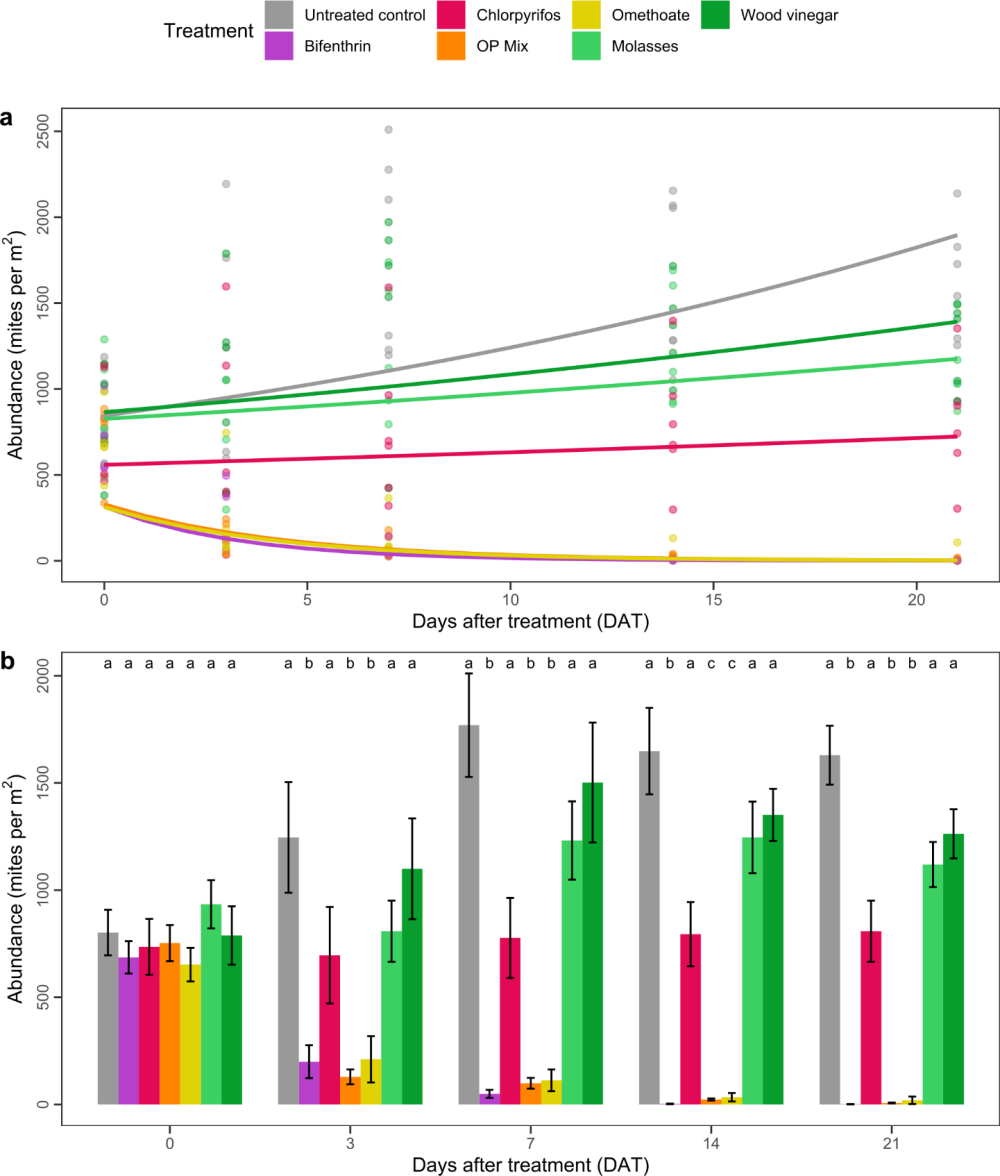
Abundances of *Halotydeus destructor* following exposure to treatments in the field. (**a**) Fitted models of abundance. Each point represents the mean abundance per plot. Lines depict the model estimated change in abundance. Points and lines are coloured by treatment (see legend). (**b**) Mean abundance (± SE) of mites. Letters above bars indicate similarity among treatments based on Tukey’s tests comparing differences between treatments within each sampling date (at a threshold of *α* = 0.05). Note, mite numbers are displayed on the non-transformed scale, but the model and post-hoc tests were run on log-transformed data

The chemical treatments varied considerably in their efficacy against *H. destructor* over the 21 days of our field trial (Fig. 1a), with model outputs showing significant differences for treatment (χ^2^ = 610, df = 6, *P* < 0.001) and sampling date (χ^2^ = 507, df = 1, *P* < 0.001), as well as their interaction (χ^2^ = 956, df = 6, *P* < 0.001). All coefficients for the repeated measures model (treatment interacting with sampling date) are reported in Table S1, with the reference level being set to the untreated control. Mite abundances increased in the untreated control (*β* = 2.93) and in the molasses, wood vinegar, and chlorpyrifos treated plots over the 21 days. In contrast, abundances decreased in the bifenthrin, omethoate, and OP Mix treatments.

Post hoc analysis revealed three key observations. Firstly, plots treated with molasses and wood vinegar had mite abundances that were not significantly different to the untreated control at each sampling date (Fig. 1b), indicating these *novel* treatments were ineffective against *H. destructor*. Secondly, chlorpyrifos was unable to effectively control *H. destructor.* At each sampling date, plots treated with chlorpyrifos had abundances that were not significantly different to plots treated with molasses and wood vinegar. Plots treated with chlorpyrifos also had mite abundances that were similar to the untreated control at 0- and 3-DAT, although lower abundances were observed at 7-, 14-, and 21-DAT (Fig. 1b). Thirdly, omethoate and the OP Mix effectively controlled *H. destructor*, resulting in similar reductions in mite abundances to those observed in plots treated with bifenthrin (the SP) at all sampling dates, with the exception of 14-DAT (Fig. 1b). By 21-DAT, mite abundances had been reduced by >98, >99, and >99% in the omethoate, OP Mix, and bifenthrin treated plots, respectively.

## Discussion

In this study, we explored the chemical responses of *H. destructor* populations to the OPs omethoate and chlorpyrifos. One of these populations was resistant, but with different levels of relative resistance, with a resistance ratio near 100 for chlorpyrifos and approximately 7 for omethoate. Given these findings, we predicted omethoate to remain relatively effective against *H. destructor* in the field due to the low observed resistance ratio, whereas we expected control of mites with chlorpyrifos to be more problematic given the higher resistance ratio for this chemical. These predictions matched the field trial data, which showed a small but persistent reduction in Res-pop abundances (compared with the untreated controls) following chlorpyrifos applications, whereas omethoate remained highly effective in controlling these mites with abundances reduced by >98% by 21-DAT, a similar level of control achieved by the SP bifenthrin. This type of finding has been observed in previous studies (e.g., Dennehy et al. 1987, Silva et al. 2011) where low-level pesticide resistance does not necessarily result in chemical control failures in the field. Importantly, previous field work by our group has shown that a high level of control is achieved when spraying insecticide-susceptible populations of *H. destructor* using the recommended field rates of both omethoate and chlorpyrifos (see Supplementary Material).

When attempting to link laboratory bioassay data with the potential for chemical control failures in the field, it is useful to undertake selection experiments to determine whether the resistance is likely to be increased to levels higher than observed in the field, and if so, how quickly that might occur. If resistance levels increase in a relatively short period of time, this would indicate a low level of resistance in the field may reflect a low frequency of resistant alleles that can be further increased by pesticide selection. Such experiments have been informative in understanding pesticide resistance in other pests (e.g., Kristensen and Jespersen 2003, Alvi et al. 2012). However, selection experiments involving *H. destructor* are not easy to perform given the inherent challenges of establishing crosses and culturing the necessary number of individuals in the laboratory (Ridsdill-Smith 1991). In small field plots, selection for resistance has demonstrated the potential for rapid change in *H. destructor*, as observed for SPs, where a single spray application of bifenthrin resulted in an abrupt increase in the level of resistance to this chemical (Umina et al. 2023). The lack of control achieved with a single application of chlorpyrifos suggests the frequency of resistance in the Res-pop was very high prior to the field trial commencing. We therefore suspect that the rapid decrease in mites in the omethoate treated plots was the result of a low resistance level, which may not necessarily increase further with selection. We note that there are other cases in the literature where, regardless of ongoing selection, the relative level of resistance remains low (e.g., Siqueira et al. 2000).

Other factors, such as chemical decay rates, can also be relevant when attempting to make comparisons of pesticide effects as we have done here. For example, in our study, a rapid decay of chlorpyrifos compared with omethoate could contribute to a shorter period of control against *H. destructor*. Organophosphates break down in the environment under the influence of sunlight, oxygen, moisture, micro-organisms and reactive soil chemicals (Ragnarsdottir 2000). Although the environmental behaviour of OPs is not clearly understood, the available information on chemical half-lives does not support more persistent effects of omethoate (or dimethoate which breaks down to omethoate) when compared with chlorpyrifos (see information about these chemicals at https://www.cdc.gov/biomonitoring and https://inchem.org/). Furthermore, our previous field trials where these two chemicals have been applied to pesticide-susceptible populations of *H. destructor* show similar persistent effects for both omethoate and chlorpyrifos (see Supplementary Material).

The specific reason(s) for the highly variable responses between omethoate and chlorpyrifos in the Res-pop mites remain unclear, although it may be linked to the underlying mechanism(s) conferring OP resistance in *H. destructor*. The resistance mechanism in this species appears to be complex and one that potentially has variable impacts on OP compounds that are structurally different, which include chlorpyrifos (*0,0*-diethyl *O*-(3.5,6-trichloropyridin-2-yl) thiophosphate) and omethoate (*O,O*-dimethyl *S*-[2-(methylamino)-2-oxoethyl] thiophosphate) (Gupta 2006) (Figure S2). Attempts by our group (and others) to elucidate the molecular mechanism conferring OP resistance in *H. destructor* have proven unsuccessful. Most recently, we have used a whole-genome pool-seq approach on several *H. destructor* populations from different geographic regions of Australia (Thia et al. 2022a). This work, which has identified multiple variants of the acetylcholinesterase gene (the neurological target of OPs), as well as variable copy numbers of these genes, has once again pointed to the potential complexity of the underpinning mechanism of resistance. Acetylcholinesterase gene copy number variation has been associated with OP resistance in other species, including *T. urticae* (Kwon et al. 2010). The complexity observed in *H. destructor* is further indicated by the fact that resistance ratios to omethoate and chlorpyrifos (as well as other OP chemicals such as malathion) are variable depending on the mite population tested. For example, somewhat different to the resistance ratios seen in this study, Umina et al. (2017) tested three resistant populations of *H. destructor* from Western Australia and found these had resistance ratios between ~13–40 for omethoate and between ~25–35 for chlorpyrifos.

Given the growing number of cases of pesticide resistance in *H. destructor* populations in Australia and the diminishing chemical control options available to farmers (Arthur et al. 2021), there is a pressing need to find alternative management tactics. Two *novel* spray treatments were trialled in this study but found to be ineffective against *H. destructor* when applied at the doses tested (4 L/ha for each product), which were informed by other studies and through discussions with local farmers. Previous research has indicated molasses might be useful in the management of *T. urticae* and *Onychiurus* spp. (Heijbroek et al. 1980; Shanks et al. 1995), whereas in other situations, it is used as an organic fertiliser, increasing the production and emission of volatile compounds by crops, which may influence pest numbers (Marangoni et al. 2004). Molasses is also included in some virus treatments to control pests (e.g., thrips; Thackeray et al. 2015) and can be added to *Bt* treatments to act as a feeding stimulant (Sabbour et al. 2012). In the former case, molasses by itself seems to have little direct impact on thrip numbers (Thackeray et al. 2015), whereas in combination with *Bt* it can increase *Helicoverpa armigera* control (Sabbour et al. 2012). Wood vinegar, or pyroligneous acid, is a by-product of the biochar process and can take many forms (Loo et al. 2008). It is typically a red-brown liquid generated from the gas and combustion of fresh wood burning under airless conditions. When the gas is cooled, it condenses into liquid, which contains acetic acid, methanol, acetone, wood oils and tars. Some wood vinegar formulations have shown potential for the control of numerous insects, as well as some mites (e.g., Yamauchi and Matsumoto 2016, Alimurung et al. 2017). Our findings suggest neither molasses nor wood vinegar are likely to be commercially viable options for broad-scale control of *H. destructor*.

In summary, we present evidence of differences in the resistance ratios for two OPs, chlorpyrifos and omethoate, in a field population of *H. destructor* with a low level of resistance. Subsequent field trials showed that the difference in resistance levels translated into a difference in the effectiveness of these two chemicals under field conditions. Given pesticide resistance in this species is expanding within Australia (Arthur et al. 2021), this finding is important from a control perspective given one of the OPs, omethoate, remained effective in controlling mites. These results suggest a close relationship between levels of resistance quantified through laboratory bioassays and the field effectiveness of pesticides. However, in the case of *H. destructor* this does not necessarily mean all field populations possessing OP resistance will respond similarly given the potentially complex nature of the underlying mechanism(s) conferring resistance to OPs. Farmers are thus unable to confidently predict the likely efficacy of different OP chemicals when spraying resistant *H. destructor* populations, pointing to the need for a fast, reliable field diagnostic to screen for resistance levels in this species.

## Electronic Supplementary Material

Below is the link to the electronic supplementary material.

Supplementary Material 1

## Data Availability

The datasets generated and analysed during the current study are available from the corresponding author on reasonable request.
