# Supplementary material for: From laboratory to field: laboratory-measured pesticide resistance reflects outcomes of field-based control in the redlegged earth mite, Halotydeus destructor
Source: Exp Appl Acarol. 2023 Mar 31;89(3-4):379–92. doi: 10.1007/s10493-023-00787-2 (PMC10167116; doi:10.1007/s10493-023-00787-2)
Supplement: Supplementary file 1 — Supplementary Material 1 [file 10493_2023_787_MOESM1_ESM.docx]

**
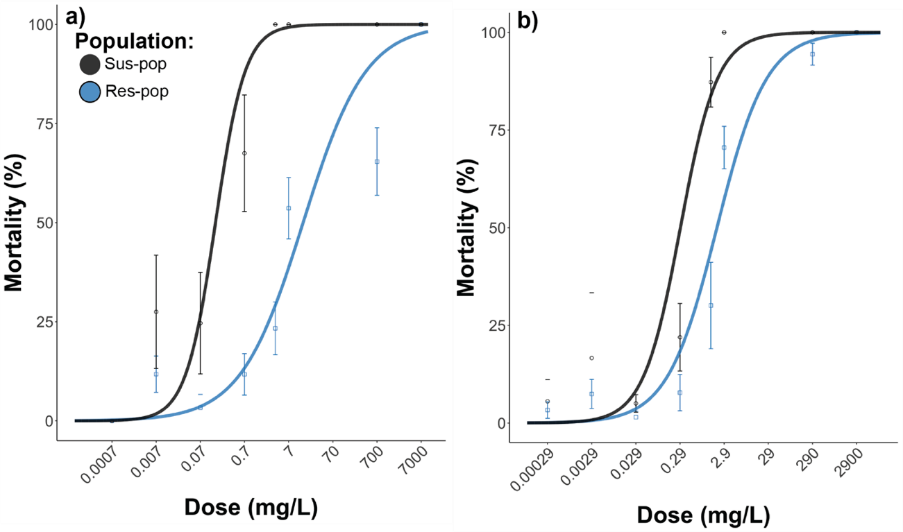
SUPPLEMENTARY MATERIAL**

**Figure S1.** Dose-response curves for *Halotydeus destructor* from the Sus-pop and Res-pop after exposure to (a) chlorpyrifos and (b) omethoate. The *x*-axis represents the dose of each active ingredient and the *y*-axis the percent mortality. Points with standard errors depict the mean mortality per dose for each population.

**Table S1.** Coefficients table for repeated measures of mite abundance following the application of different treatments in the field

| **Coefficient** | **Estimate (*β*)** | **SE** | **df** | **t** | ***P*** |
| --- | --- | --- | --- | --- | --- |
| Intercept | 2.93 | 0.09 | 60 | 32.89 | < 0.001 |
| Bifenthrin | -0.43 | 0.13 | 60 | -3.41 | 0.001 |
| Chlorpyrifos | -0.18 | 0.13 | 60 | -1.43 | 0.16 |
| OP Mix | -0.41 | 0.13 | 60 | -3.26 | 0.002 |
| Omethoate | -0.43 | 0.13 | 60 | -3.43 | 0.001 |
| Molasses | -0.01 | 0.13 | 60 | -0.07 | 0.94 |
| Wood vinegar | 0.01 | 0.13 | 60 | 0.08 | 0.94 |
| Sampling date | 0.02 | 0.01 | 753 | 3.31 | 0.001 |
| Bifenthrin:sampling date | -0.15 | 0.01 | 753 | -20.51 | < 0.001 |
| Chlorpyrifos:sampling date | -0.01 | 0.01 | 753 | -1.59 | 0.11 |
| OP Mix:sampling date | -0.12 | 0.01 | 753 | -16.19 | < 0.001 |
| Omethoate:sampling date | -0.12 | 0.01 | 753 | -16.54 | < 0.001 |
| Molasses:sampling date | -0.01 | 0.01 | 753 | -1.26 | 0.21 |
| Wood vinegar:sampling date | -0.01 | 0.01 | 753 | -0.92 | 0.36 |

Note: Model fitted the interaction between ‘treatment’ and ‘sampling date’, with the untreated control set as the reference level. Terms for other treatment levels represent their deviation from the untreated control.

**Field trial 1. Efficacy of omethoate – Heathcote, Victoria**

The efficacy of a single rate of Le-mat (290 g/L omethoate; field rate = 100 mL per 100 L/ha) against *H. destructor* was examined in a large-scale field trial near Heathcote (Victoria, Australia). Le-mat was tested alongside Fastac Duo (100 g/L alpha-cypermethrin; field rate = 100 mL per 100 L/ha), a pyrethroid insecticide commonly used to control *H. destructor,* and an untreated control. This trial was undertaken in a long-term pasture paddock, using a randomised complete block design with four replicate blocks containing plots 10 × 10 m in size.

Following trial setup, all plots were sampled for mite numbers via suction using a Stihl blower vacuum with a 100-micron steel sieve fitted to the end of the vacuum spout. A defined area within a 0.09 m^2^ frame was vacuumed and the contents were transferred to a sorting tray where *H. destructor* were identified and counted. Four replicate samples were collected within each plot. Plots were then sprayed with the assigned treatment using UniBoom trailing boom spray (model 600L TR) (see Table S2). Mite numbers within each plot were subsequently sampled 2 days after treatment (DAT), 7-DAT, 12-DAT and 28-DAT using the methods described above.

**Table S2.** Site and spray information for field trial 1 conducted at Heathcote, Victoria

| **Location** | Heathcote, Victoria |
| --- | --- |
| **GPS co-ordinates** | -37.020, 144.671 |
| **Paddock history** | Ryegrass and clover pasture for >5 years |
| **Spray method** | Trailing boom spray, UniBoom*^®^* model 600L TR |
| **Spray volume** | 100 L/ha |
| **Spray pressure** | 300 kPa |
| **Nozzle type** | TeeJet Flatfan 02, 0.87 L/min |
| **Vehicle speed** | 10 km/h |

Prior to analysis, mite count data was converted to number per metre square and log(*n* + 1)-transformed. Data was then checked for normality using the Kolmogorov-Smirnov test (normal distribution) and Levene’s test (homogeneity of variances) following Sokal and Rohlf (1995). Differences in mite numbers between treatments at each sampling date (0-DAT, 2-DAT, 7-DAT, 12-DAT and 28-DAT) were then determined using one-way ANOVAs and Tukey’s-*b* post hoc tests. Analyses were conducted using the software SPSS Statistics (v.26).

Mite pressure was high, but statistically similar, across all plots immediately prior to chemical application in field trial 1 (i.e., 0-DAT). Subsequent reductions in mite numbers were demonstrated by the application of both omethoate and alpha-cypermethrin (Table S2). By 2-DAT, omethoate had statistically fewer mites than alpha-cypermethrin, which had statistically fewer mites than the untreated control. This pattern continued at 7-DAT and 12-DAT. By 28-DAT, both omethoate and alpha-cypermethrin had far fewer mites than the untreated control plots (Table S3).

**Table S3.** Average number of *Halotydeus destructor* per m^2^ in field trial 1 conducted at Heathcote, Victoria. Different letters indicate significantly different means

| Treatment | 0-DAT | | 2-DAT | | 7-DAT | | 12-DAT | | 28-DAT | |
| --- | --- | --- | --- | --- | --- | --- | --- | --- | --- | --- |
| Untreated control | 5411 | a | 11178 | a | 7756 | a | 3233 | a | 4400 | a |
| Le-mat^®^ | 8389 | a | 1189 | c | 944 | c | 44 | c | 33 | b |
| Fastac^®^ Duo | 7922 | a | 3922 | b | 3256 | b | 744 | b | 44 | b |
| *P* | 0.28 | | < 0.001 | | < 0.001 | | < 0.001 | | < 0.001 | |
| F_2,45_ | 1.32 | | 50.53 | | 28.68 | | 60.43 | | 110.82 | |

**Field trial 2. Efficacy of chlorpyrifos – Inverleigh, Victoria**

The efficacy of a single rate of Lorsban (chlorpyrifos 500 g/L; field rate = 140 mL per 100 L/ha) against *H. destructor* was examined in a large-scale field trial near Inverleigh (Victoria, Australia). Lorsban was tested alongside Karate Zeon (lambda-cyhalothrin 250 g/L; field rate = 9 mL per 100L/ha), a pyrethroid insecticide commonly used to control *H. destructor,* and an untreated control. This trial was undertaken in a recently sown oat (*Avena sativa*) crop, using a randomised complete block design with four replicate blocks containing plots 10 × 8 m in size.

Following trial setup, all plots were sampled for mite numbers via suction using a Stihl blower vacuum with a 100-micron steel sieve fitted to the end of the vacuum spout. A defined area within a 0.09 m^2^ frame was vacuumed and the contents were transferred to a sorting tray where *H. destructor* were identified and counted. Four replicate samples were collected within each plot. Plots were then sprayed with the assigned treatment using UniBoom trailing boom spray (model 600L TR) (see Table S4). Mite numbers within each plot were subsequently sampled 3-DAT, 7-DAT, 14-DAT and 28-DAT using the methods described above.

**Table S4.** Site and spray information for field trial 2 conducted at Inverleigh, Victoria

| **Location** | Inverleigh, Victoria |
| --- | --- |
| **GPS co-ordinates** | -38.134, 144.045 |
| **Paddock history** | Oats (*Avena sativa*) |
| **Spray method** | Trailing boom spray, UniBoom^®^ model 600L TR |
| **Spray volume** | 100 L/ha |
| **Spray pressure** | 300 kPa |
| **Nozzle type** | TeeJet Air Induction XR Flat Spray (AIXR11002) |
| **Vehicle speed** | 10 km/h |

Prior to analysis, mite count data was converted to number per metre square and log(*n* + 1)- transformed. Data was then checked for normality using the Kolmogorov-Smirnov test (normal distribution) and Levene’s test (homogeneity of variances) following Sokal and Rohlf (1995). Differences in mite numbers between treatments at each sampling date (0-DAT, 3-DAT, 7-DAT, 14-DAT and 28-DAT) were then determined using one-way ANOVAs and Tukey’s-*b* post hoc tests. Analyses were conducted using the software SPSS Statistics (v.26).

Mite pressure was relatively low, but statistically similar, across all plots immediately prior to chemical application in field trial 2 (i.e., 0-DAT). Subsequent reductions in mite numbers were demonstrated by the application of both chlorpyrifos and lambda-cyhalothrin (Table S4). At 3-DAT and 7-DAT, both chlorpyrifos and lambda-cyhalothrin had far fewer mites than the untreated control plots. At both 14-DAT and 28-DAT, lambda-cyhalothrin had statistically fewer mites than chlorpyrifos, which had statistically fewer mites than the untreated control (Table S5).

**Table S5.** Average number of *Halotydeus destructor* per m^2^ in field trial 2 conducted at Inverleigh, Victoria. Different letters indicate significantly different means at each sampling date (at a threshold of *α* = 0.05, Tukey’s-*b* post hoc test)

| Treatment | 0-DAT | | 3-DAT | | 7-DAT | | 14-DAT | | 28-DAT | |
| --- | --- | --- | --- | --- | --- | --- | --- | --- | --- | --- |
| Untreated control | 788 | a | 387 | a | 430 | a | 472 | a | 581 | a |
| Lorsban^®^ | 831 | a | 101 | b | 73 | b | 62 | b | 162 | b |
| Karate Zeon^®^ | 826 | a | 103 | b | 69 | b | 20 | c | 20 | c |
| *P* | 0.75 | | < 0.001 | | < 0.001 | | < 0.001 | | < 0.001 | |
| F_2,45_ | 0.29 | | 19.16 | | 26.68 | | 50.46 | | 22.85 | |

**(a)**

**
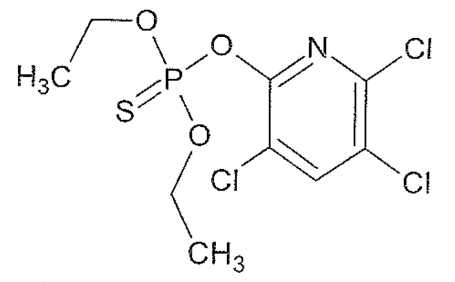
**

**(b)**

**
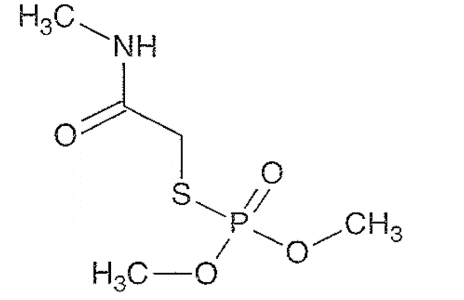
**

**Figure S2.** Chemical structure of the organophosphorus insecticides (a) chlorpyrifos and (b) omethoate. Figures reproduced from Gupta (2006).

**References**

Gupta, R. C. 2006. Classification and uses of organophosphates and carbamates, pp. 5–24. *In* R. C. Gupta, Toxicology of organophosphate and carbamate compounds. Academic Press, Kentucky, USA.

Sokal, R. R., and F. J. Rohlf. 1995. Biometry: The principles and practice of statistics in biological research, 3rd edition. W.H. Freeman. New York, USA.
